# Leveraging access to technology and enhanced surgical technique (LATEST) in laparoscopic bile duct exploration (LBDE)

**DOI:** 10.1007/s00464-022-09667-z

**Published:** 2022-10-17

**Authors:** Lalin Navaratne, Jasim Al-Musawi, Kritchai Vutipongsatorn, Alberto Martinez Isla

**Affiliations:** 1 FRCS, Department of Upper GI Surgery, Northwick Park Hospital, St Mark’s Hospitals, London North West University Healthcare NHS Trust, London, UK; 2 Emergency Surgery Unit, Northwick Park Hospital, St Mark’s Hospitals, London North West University Healthcare NHS Trust, London, UK

**Keywords:** Laparoscopic bile duct exploration, Laparoscopic common bile duct exploration, Choledocholithiasis, Common bile duct stones, CBD stones, Leveraging access to technology and enhanced surgical techniques

## Abstract

Single-stage management of choledocholithiasis with concomitant gallstones consists of performing either laparoscopic bile duct exploration (LBDE) or intra-operative endoscopic retrograde cholangiopancreatography at the same time as laparoscopic cholecystectomy. Transductal LBDE is associated with significantly higher post-operative morbidity, longer operative times and longer hospital stay when compared to transcystic LBDE. The aim of this study was to report the transcystic exploration rate and post-operative outcomes from LBDE before and after implementation of the LATEST (Leveraging Access to Technology and Enhanced Surgical Technique) principles. Methods: A retrospective review of 481 consecutive patients between February 1998 and July 2021 was performed. Patients were assigned into two groups determined by whether they were operated before or after the implementation of LATEST. Data collected included pre-operative demographic information, medical co-morbidity, pre-operative investigations, and intra-operative findings (including transcystic exploration rate, negative choledochoscopy rate, use of holmium laser lithotripsy and operative time). Outcomes of this study were the transcystic exploration rate, stone clearance rate, conversion to open surgery, post-operative morbidity and mortality, and length of post-operative hospital stay. Results: The *pre-LATEST* group contained 237 patients and the *LATEST* group comprised of 244 patients. Ultra-thin choledochoscopes and holmium laser lithotripsy were used more frequently in the *LATEST* group (41.4% and 18.4%, respectively). Enhanced surgical techniques (correction of the cystic duct-CBD junction and the trans-infundibular approach) were also performed more frequently in the *LATEST* group. More patients in the *LATEST* group received transcystic LBDE (86.1% vs 11.0%, *p* < 0.0001). The *LATEST* group had significantly higher stone clearance rates (98.8% vs 93.7%, *p* = 0.0034), reduced post-operative morbidity and shorter post-operative hospital stay (4 days vs 1 day, *p* < 0.0001). Conclusions: LATEST describes four key factors that can be used when performing LBDE. The adoption of LATEST in LBDE is associated with an increased stone clearance, a higher transcystic exploration rate and reduced post-operative morbidity.

**Supplementary Information:**

The online version contains supplementary material available at 10.1007/s00464-022-09667-z.

Common bile duct (CBD) stones are present in up to 15% of patients with symptomatic gallstones [1, 2]. Single-stage management of choledocholithiasis with concomitant gallstones consists of performing either laparoscopic bile duct exploration (LBDE) or intra-operative endoscopic retrograde cholangiopancreatography (ERCP) at the same time as laparoscopic cholecystectomy (LC). In the United Kingdom, surgical clearance of the bile duct, if needed, is recommended at the time of laparoscopic cholecystectomy provided the laparoscopic expertise are available [3]. In a recent meta-analysis including nearly three thousand patients, LC-intra-operative ERCP and LC-LBDE exhibited similar efficacies when clearance rate, overall post-operative complications, conversion to laparotomy and operative time were compared [4]. When compared to two-staged approaches (LC with either pre- or post-operative ERCP), single-stage management has been proven to be the superior strategy [5]. Furthermore, several studies have demonstrated that single-stage management is associated with reduced hospital stay and hospital costs [6–9]. Currently, however, there is no evidence to demonstrate that single-stage management results in less post-operative morbidity [8–10]. We have previously provided a possible explanation for this finding [11, 12]. The pooled patients who underwent LBDE within historical randomised controlled trials and included in more recent systematic reviews mostly received transductal LBDE via choledochotomy, with less than one-third having received an attempted transcystic LBDE. There is overwhelming evidence that transductal LBDE is associated with significantly higher post-operative morbidity, mainly in the form of bile leak (and post-operative pancreatitis if the bile duct was closed over an antegrade stent), longer operative times and longer hospital stay [12–15]. When approximately only a third of patients undergoing LBDE within prospective RCTs received transcystic LBDE, it is likely that the pooled morbidity associated with bile leak (and pancreatitis) from the LBDE arm is an overestimate of the true morbidity associated with transcystic LBDE. For surgeons performing LBDE, the primary goal should therefore be to achieve high rates of transcystic exploration, and performing choledochotomy for transductal access only when it is unavoidable and absolutely necessary.[12].

We recently proposed the concepts of *Biliary Surgery 2.0* and Leveraging Access to Technology and Enhanced Surgical Technique (LATEST) in LBDE (Fig. 1) [16, 17]. *Leveraging access to technology* includes using thinner and more flexible choledochoscopes combined with fragmentation techniques such as laser or electrohydraulic lithotripsy. *Enhanced surgical technique* refers to full mobilisation of the gallbladder followed by complete dissection of the cystic duct to the cysticocholedochal junction and the trans-infundibular approach, which we have previously described, and is indicated when Calot’s triangle cannot be safely dissected due to a ‘frozen’ hepatic hilum secondary to severe inflammation and/or fibrosis [18]. The aforementioned ‘pillars’ of LATEST were all implemented around the same time within 2014. The aim of this study is to report the transcystic exploration rate and post-operative outcomes from LBDE before and after implementation of the LATEST principles.

**Fig. 1:**
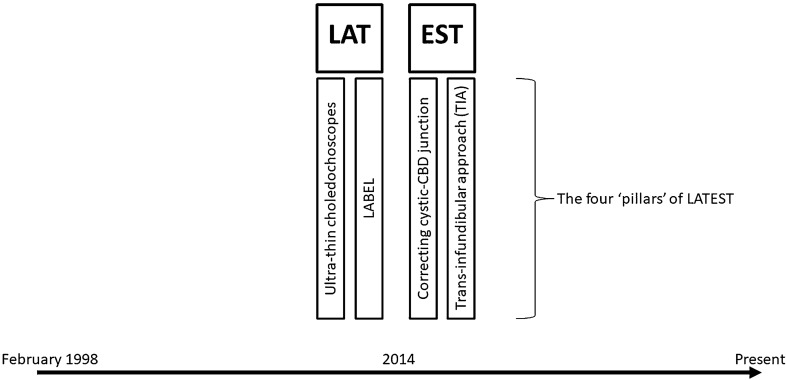
Four ‘pillars’ of Leveraging Access to Technology and Enhanced Surgical Technique (LATEST)

## Methods

### Patients

A retrospective review of a prospectively collected database of 481 consecutive patients who underwent LBDE at a single centre between February 1998 and July 2021 was performed. Ethical approval was not required for this type of study. All operations were performed or supervised by the senior surgeon (AI). After review of medical records, all patients were assigned into two groups determined by whether they were operated before or after the implementation of LATEST. Patients who underwent LBDE between February 1998 and February 2014 were grouped as ‘*pre-LATEST*’, whereas patients who had surgery after this date were classified as ‘*LATEST*’. All patients were assessed with pre-operative liver function tests (LFTs) and abdominal imaging. Data collected included pre-operative demographic information, medical co-morbidity, pre-operative investigations, intra-operative findings (including transcystic exploration rate, negative choledochoscopy rate, use of holmium laser lithotripsy and operative time) and post-operative outcomes. Clinical presentation was classified as pancreatitis, hyperbilirubinaemia, deranged LFTs and dilated CBD (≥ 8 mm on pre-operative imaging). Pancreatitis was diagnosed as per the Atlanta classification [19]. Patients were classified as ‘hyperbilirubinaemia’ if the bilirubin was more than two times the upper limit of normal irrespective of the liver enzymes (alanine aminotransferase [ALT] and alkaline phosphatase [ALP]). Those patients with abnormal liver enzymes but bilirubin within the normal range or less than two times the upper limit of normal were classified as ‘deranged LFTs’ irrespective of CBD diameter. Patients were classified as ‘dilated CBD’ if this was found on pre-operative imaging along with normal bilirubin and LFTs.

Outcomes of this study were the transcystic exploration rate, stone clearance rate, conversion to open surgery, post-operative morbidity and mortality and length of post-operative hospital stay. Stone clearance was confirmed by choledochoscopy or completion cholangiogram if a proximal view of the intra-hepatic ducts was not obtained intra-operatively. Failure of LBDE was defined as patients who required conversion to open surgery or those with retained CBD stones known intra-operatively (failure to extract stones during procedure) or diagnosed within 12 months of surgery. Patients with retained stones were managed with post-operative ERCP. Bile leaks were graded according to the 2011 International Study Group of Liver Surgery (ISGLS) classification [20]. Length of post-operative hospital stay was chosen instead of total length of hospital stay because patients with acute cholecystitis, obstructive jaundice or pancreatitis were often admitted under the emergency surgery service and remained as inpatients until their operation could be scheduled on the next dedicated biliary list.

### Surgical technique

The surgical approach to the bile duct was either transductal via choledochotomy or transcystic and has previously been described [12, 21–23]. In the *LATEST* group, the transcystic route was the approach of choice and attempted first routinely, whereas in the *pre-LATEST* group, the transcystic route was attempted only in selected cases (usually when the anatomy was favourable and there was a dilated cystic duct).

### Laparoscopic cholecystectomy and intra-operative cholangiogram

Patients were positioned using the French technique for all operations, with the surgeon standing between the legs and the assistant to the left side of the patient [24]. Laparoscopic port placement was performed in accordance with our previously described technique [18]. In brief, Veress needle insufflation was followed by insertion of a 12-mm trocar in the left upper quadrant using an optical entry system. A 5-mm 30° laparoscope was inserted under direct vision, adjacent to the distal end of the falciform ligament, some 12–15 cm below the xiphoid process to allow optimal views of the CBD and Calot’s triangle. A Nathanson’s liver retractor was used to maintain the operative view when necessary. Dissection of Calot’s triangle to achieve the critical view of safety and cystic artery ligation was performed in the standard way. When indicated, intra-operative cholangiogram (IOC) was performed using a cholangiocatheter (5F ureteric catheter, open-end straight tip, 70-cm long, Cook Medical, Bloomington, IN, USA) placed into the cystic duct using a Horner needle (Steriseal Horner™, Optech Diagnostic & Surgical, East Melbourne, Australia) or a single-use ENT suction tube (NETWORK ENT®, Network Medical Products Ltd, North Yorkshire, UK) for transabdominal access. The cholangiocatheter was railroaded over a guidewire (PTFE Wire Guide with 3-cm flexible tip, 0.035" diameter, 145-cm long, Cook Medical, Bloomington, IN, USA) when primary intubation of the cystic duct was difficult. In complex cases where the hilum was ‘frozen’ with inflammation and/or fibrosis, the anatomy was clarified with intra-operative cholangiogram and/or use of the trans-infundibular approach (TIA) to the CBD technique [18].

### Transductal common bile duct exploration

Our technique for transductal LBDE has previously been described. [12] The CBD diameter was measured intra-operatively using a tape measure and choledochotomy was only performed if the diameter was > 8 mm. When choledochotomy was required, a longitudinal incision was performed using laparoscopic scissors or Berci knife. Choledochoscopy was performed using a 3- or 5-mm choledochoscope depending on availability and biliary tree anatomy. Introduction of the 3-mm choledochoscope required insertion of a 9.5–12F sheath (Flexor Ureteral Access Sheath, 45-cm long, Cook Medical, Bloomington, IN, USA), or another 5-mm port in the right upper quadrant for the 5-mm choledochoscope. The choledochoscope was passed freely through the choledochotomy into the bile duct for proximal and distal choledochoscopy. Standard retrieval techniques were then used for stone extraction when required. After February 2014, the LABEL technique using holmium laser lithotripsy to fragment large and/or impacted stones was implemented at our centre. [25, 26] When required, the fibre-optic holmium laser (200–365 μm, ScopeSafe™, Optical Integrity, FL, USA) was introduced through the working channel of the choledochoscope. The laser energy setting used initially was 0.5 J (with a frequency of 20 Hz) and increased incrementally as required to achieve fragmentation. Lithotripsy was achieved by aiming the light diode at the stone and activating the laser. Laser safety precautions were strictly adhered to at all times. After clearance of the common bile duct, the choledochotomy was closed over an antegrade stent, closed using a T-tube or closed primarily (with or without a transcystic drain). At the beginning of the series, closure with T-tube was the favoured technique until 2001. Over the next decade, closure over an antegrade stent was the preferred method but was abandoned due to the increased incidence of post-operative pancreatitis. From 2011, primary closure of choledochotomy was the first line approach when transductal LBDE was required. This was performed by placing a retracting stay suture at the cranial end of the incision and closing the incision primarily using continuous 5–0 Vicryl (Ethicon, Somerville, NJ, USA) on a curved round bodied needle starting from the caudal end.

### Transcystic common bile duct exploration

Prior to 2014, intra-operative cholangiogram and transcystic access for LBDE was attempted with the gallbladder still attached to the liver. Transcystic LBDE would generally only be attempted if there was favourable anatomy, and the cystic duct was dilated. Even then, the opening of the cystic duct would have to be gently dilated using Maryland or Johan forceps in order to accommodate the 5-mm flexible choledochoscope. After 2014, once the critical view of safety had been achieved and the cystic artery ligated, the gallbladder was fully mobilised from the liver. The cystic duct was carefully skeletonised down to its junction with the bile duct, and the infundibular-cystic duct junction was then retracted through the abdominal wall using an Endoloop (Ethicon, New Brunswick, New Jersey, USA) and Endo Close™ (Covidien, Mansfield, Massachusetts, USA) to create the optimal cysticocholedochal angle. [12] Since 2014, this technique has become the standard approach when IOC and transcystic LBDE have been performed. Choledochoscopy was performed via the cystic duct using a 3- or 5-mm choledochoscope depending on cystic duct diameter and equipment availability. The reusable 3-mm choledochoscope was not always available due to equipment being sent off site for sterilisation, maintenance or repair, and therefore after 2017, we have routinely been using disposable, single-use choledochoscopes. The two most commonly used endoscopes were the PUSEN (Zhuhai PUSEN Medical Technology Company Ltd, China) and SpyGlass™ Discover (Boston Scientific, Marlborough, MA, USA). Introduction of the 3-mm choledochoscope required insertion of a 9.5–12F sheath (Flexor Ureteral Access Sheath, 45-cm long, Cook Medical, Bloomington, IN, USA), or another 5-mm port in the right upper quadrant for the 5-mm choledochoscope. Standard retrieval techniques were then used for stone extraction when required. If large and/or impacted stones were encountered, the LABEL technique was used (after February 2014) to fragment and extract the stone(s), avoiding failure and/or choledochotomy. Stone clearance was confirmed by proximal and distal choledochoscopy. Proximal choledochoscopy of the intra-hepatic ducts was performed using the ‘wiper blade manoeuvre’ [27]. If this was not feasible, a completion cholangiogram was performed to confirm clearance of the bile duct.

### Leveraging access to technology and enhanced surgical technique (LATEST)

**Fig. 2:**
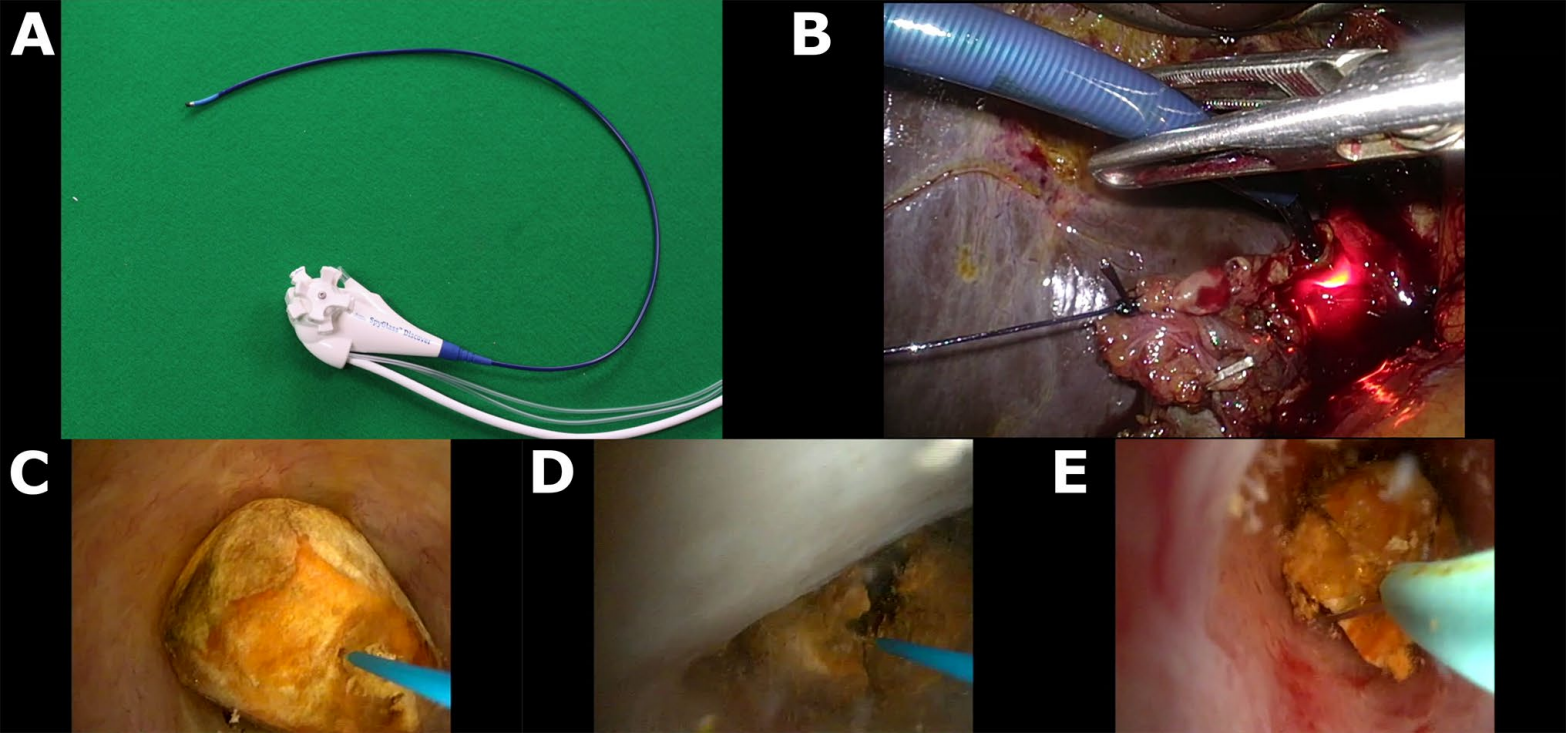
Leveraging Access to Technology (LAT). **A**, an example of an ultra-thin choledochoscope (pictured: SpyGlass™ Discover, Boston Scientific, Marlborough, MA, USA). **B**, insertion of a 9.5F access sheath for transcystic access with ultra-thin choledochoscope intubating the cystic duct. **C**, large common bile duct stone with laser probe in position to perform lithotripsy. **D**, fragmentation using laser lithotripsy. E, removal of fragments with basket

**Fig. 3:**
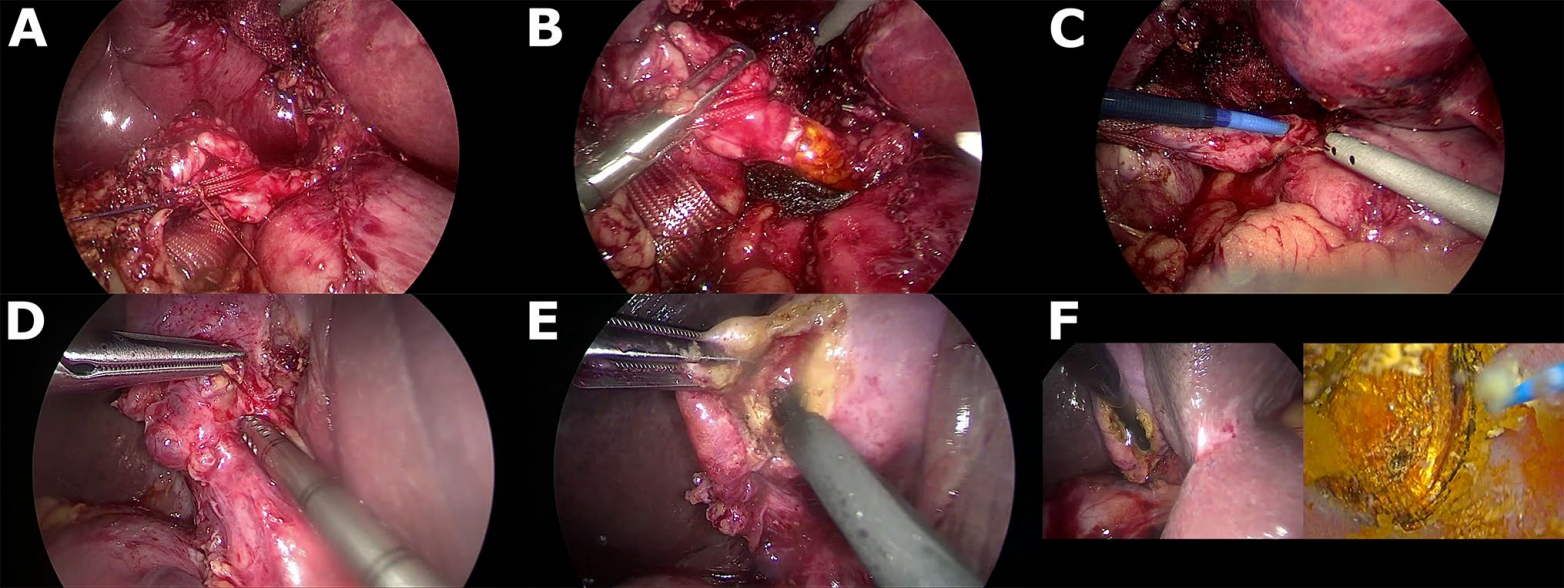
Enhanced Surgical Technique (EST). **A**-**C**, correction of the cysticocholedochal angle. **D**-**F**, Trans-infundibular Approach (TIA) to the common bile duct

LATEST was defined by four factors that have changed practice and contributed to the evolution of LBDE at our institution since 2014 (Fig. 1). *(1) Ultra-thin (*~ *3 mm) choledochoscopes* (supplementary file: video 1). A non-dilated cystic duct would usually preclude transcystic access using a 5-mm choledochoscope. The use of thinner, more flexible and readily available single-use choledochoscopes enabled transcystic access even when the cystic duct was not dilated (Fig. 2A). A 9.5–12F ureteral access sheath (Flexor Ureteral Access Sheath, Cook Medical, Bloomington, IN, USA) was routinely used to intubate and gently dilate the cystic duct over a PTFE guidewire (0.035-inch diameter, 145-cm length, 3-cm flexible tip, Cook Medical, Bloomington, IN, USA) (Fig. 2B). *(2) Lithotripsy-Assisted Bile duct Exploration by Laparoendoscopy (LABEL)* (supplementary file: video 2). We have previously shown that the use of lithotripsy techniques as an adjunct to LBDE has increased transcystic exploration rates (Fig. 2C-E) [26]. *(3) Correction of the cysticocholedochal angle* (supplementary file: video 3). Following dissection of Calot’s triangle, having satisfactorily achieved the critical view of safety and ligation of the cystic artery, the gallbladder was fully mobilised from the liver. The cystic duct was completely skeletonised to the cysticocholedochal junction and the infundibular-cystic duct junction retracted through the abdominal wall (Fig. 3A-C). *(4) Trans-infundibular approach (TIA) to the CBD* (supplementary file: video 4). A frozen hepatic hilum secondary to chronic inflammation and fibrosis is encountered in approximate 5% of patients who are undergoing LBDE (Fig. 3D) [18]. This scenario would usually result in failure of LBDE due to conversion to open surgery and/or abandoning bile duct exploration in favour of subtotal cholecystectomy and post-operative ERCP. TIA to the CBD is an enhanced surgical technique that allows safe cannulation of the cystic duct via the infundibulum to perform cholangiography and/or trans-infundibular (transcystic) choledochoscopy (Fig. 3E-F) [18].

### Statistical analysis

Normally distributed continuous data are reported as mean (with standard deviation), whereas skewed or ordinal data are reported as medians (with inter-quartile range). Categorical variables are expressed as number and/or frequencies (%). Statistical analysis of categorical data was performed using Chi-square or Fisher’s exact tests, while the unpaired t test or Mann–Whitney test were used for parametric or nonparametric continuous data where appropriate. A *p* value of < 0.05 was considered statistically significant. Data for this study were stored and collated using Microsoft Excel (Microsoft, Redmond, WA, USA) and GraphPad Prism 9 (GraphPad, Software Inc, La Jolla, CA, USA) statistical package.

## Results

For the study period, 481 patients were eligible and included for analysis. The *pre-LATEST* group contained 237 patients who underwent LBDE prior to February 2014 and the *LATEST* group comprised of 244 patients who were operated on after February 2014. The two groups were similar in age and gender (Table 1). The *pre-LATEST* group had significantly higher pre-operative American Society of Anaesthesiologists (ASA) scores when compared to the *LATEST* group (*p* = 0.0113). This was likely due to proportionately more patients having ischaemic heart disease and hypertension, however, only the difference in hypertension between the two groups reached significance. A related finding was that significantly more patients were taking anti-platelet medication in the *pre-LATEST* group (*p* = 0.0436). All other major co-morbidities were similar between the two groups. The proportion of patients presenting with deranged LFTs and pancreatitis were similar between the two groups. More patients in the *pre-LATEST* group presented with hyperbilirubinaemia (51.9% vs 30.3%, *p* < 0.0001); however, pre-operative bilirubin was not significantly different between the two groups (52 mmol/L vs 41 mmol/L, *p* = 0.0828). Pre-operative ALP, but not ALT, and pre-operative CBD diameter was also significantly higher in the *pre-LATEST* group. Within the whole series, 91 (19%) patients had undergone failed ERCP prior to surgery. Failure to cannulate the CBD was the reason for failure in 19 (4%) patients, whereas in 72 (15%) patients there was failure of stone extraction. There were significantly more patients who underwent a failed pre-operative ERCP prior to LBDE within the *pre-LATEST* group. From the 48 (20.3%) patients within the *pre-LATEST* group who had a pre-operative ERCP with failed stone extraction, only three (6%) went on to have transcystic LBDE. In contrast, within the *LATEST* group, 24 (9.8%) patients had an unsuccessful pre-operative ERCP due to failed stone extraction, however, 16 (67%) went on to have transcystic clearance laparoscopically (*p* < 0.0001).

**Table 1:** Pre-LATEST vs LATEST

	Pre-LATEST (*n* = 237)	LATEST (*n* = 244)	*p* value
Age (median)	58	56	0.3816
Gender (% male)	29.5	34.0	0.3277
*Pre-operative fitness for surgery (%)*			
ASA 1	38.5	37.5	**0.0113**
ASA 2	40.6	53.0	
ASA 3	20.8	9.5	
*Medical co-morbidity (%)*			
Anti-platelet medication	7.6	3.3	**0.0436**
Ischaemic heart disease	6.8	3.3	0.0951
Hypertension	41.2	26.3	**0.0088**
Atrial fibrillation	1.0	2.5	0.6779
Congestive cardiac failure	0	0.8	> 0.9999
Stroke/Transient ischaemic attack	3.1	2.1	0.6927
Chronic obstructive pulmonary disease	1.0	3.7	0.2926
Asthma	6.2	7.0	> 0.9999
Pulmonary embolus/DVT	0	1.6	0.5809
Diabetes Mellitus	12.4	9.8	0.5581
Chronic kidney disease	2.1	1.2	0.6254
Hypothyroidism	7.2	6.2	0.8073
*Clinical Presentation (%)*			
Dilated CBD	3.0	16.4	** < 0.0001**
Deranged LFTs	27.0	34.8	0.0757
Hyperbilirubinaemia	51.9	30.3	** < 0.0001**
Pancreatitis	18.1	18.4	> 0.9999
*Pre-operative LFTs*			
Bilirubin (mmol/L)	52	41	0.0828
ALP (U/L)	387	227	** < 0.0001**
ALT (U/L)	195	198	0.9187
*Pre-operative imaging*			
Median CBD diameter, mm (IQR)	12 (10 – 15)	10 (8 – 13)	** < 0.0001**
*Pre-operative ERCP (%)*			
Not performed	72.2	87.7	** < 0.0001**
Diagnostic	0.8	0.8	> 0.9999
False negative	0	0.4	> 0.9999
Failed ERCP: failure to cannulate	6.8	1.2	**0.0019**
Failed ERCP: failure to extract stones	20.3	9.8	**0.0014**

Pre-operative data. *ASA* American Society of Anesthesiologists; *DVT* deep vein thrombosis; *CBD* common bile duct; *LFT* liver function tests; *ALP* alkaline phosphatase; *ALT* alanine aminotransferase; *IQR* inter-quartile range; *ERCP* endoscopic retrograde cholangiopancreatography

Bold text denotes *p* < 0.05

Intra-operative data for the *pre-LATEST* and *LATEST* groups are summarised in Table 2. Negative CBD exploration (negative choledochoscopy) occurred almost twice as frequently in the *LATEST* group when compared with the *pre-LATEST* group (12.7% vs 22.5%, *p* = 0.0058). Although the median number of stones were similar between the two groups, the median size of the largest stone was slightly larger in the *pre-LATEST* group (7 mm vs 6 mm, *p* = 0.0024). Due to uptake and availability at our institution, ultra-thin choledochoscopes were also used much more frequently in the *LATEST* group (0% vs 41.4%, *p* < 0.0001). Of those that underwent transcystic LBDE, an ultra-thin choledochoscope was not used in any patients within the *pre-LATEST* group (*n* = 26), but was used in 210 (46.7%) patients in the *LATEST* group (*p* < 0.0001). Within the last 100 patients of the series, 94 underwent transcystic LBDE, and of those, 67 (71%) patients underwent transcystic exploration with an ultra-thin choledochoscope. Similarly, the LABEL technique was only available at our institution after implementation of the LATEST principles. LABEL was used in 45 (18.4%) patients within the *LATEST* group compared to none in the *pre-LATEST* group (*p* < 0.0001). Enhanced surgical techniques were also performed more frequently in the *LATEST* group. Correction of the cystic duct-common bile duct junction, using the technique as described, was implemented prior to 2014. It was adopted in just 38 (16.0%) patients within the *pre-LATEST* group compared to all 244 patients within the *LATEST* group (*p* < 0.0001). TIA to the CBD was only described after February 2014 and was used in 13 (5.3%) patients within the *LATEST* group. In all these patients, a complete (total) cholecystectomy was achieved after clarification of the anatomy. The approach to the CBD was significantly different between the two groups, with the transcystic route being achieved in just 26 (11%) patients in the *pre-LATEST* group compared to 210 (86.1%) patients in the *LATEST* group (*p* < 0.0001). A direct consequence of the reduction in transductal exploration was observed as reduced requirement for T-tube drainage (17.7% vs 3.3%, *p* < 0.0001), antegrade stent use (59.1% vs 4.9%, *p* < 0.0001) and intra-abdominal drain use (92.8% vs 30.3%, *p* < 0.0001) within the *LATEST* group. Operative times were similar between the two groups (*p* = 0.3647).

**Table 2:** Pre-LATEST vs LATEST

	Pre-LATEST (*n* = 237)	LATEST (*n* = 244)	*p* value
Negative choledochoscopy (%)	30 (12.7)	55 (22.5)	**0.0058**
Median number of stones (IQR)	1 (1 – 3)	1 (1 – 3)	0.0809
Median size of largest stone, mm (IQR)	7 (5 – 11)	6 (4 – 10)	**0.0024**
Intra-abdominal drain	220 (92.8)	74 (30.3)	** < 0.0001**
Median operative time, min (IQR)	115 (90 – 146)	117 (91 – 154)	0.3647
*Leveraging Access to Technology (%)*			
LABEL	0 (0)	45 (18.4)	** < 0.0001**
Ultra-thin (3 mm) choledochoscopes	0 (0)	101 (41.4)	** < 0.0001**
*Enhanced Surgical Technique (%)*			
Correction of the cysticocholedochal angle	38 (16.0)	244 (100)	** < 0.0001**
Trans-infundibular approach (TIA)	0 (0)	13 (5.3)	**0.0002**
*Approach to CBD (%)*			
Transductal (via choledochotomy)	211 (89.0)	34 (13.9)	** < 0.0001**
3-mm choledochoscopy	0 (0)	3 (8.8)	**0.0025**
5-mm choledochoscopy	211 (100)	31 (91.2)	**0.0025**
Transcystic	26 (11.0)	210 (86.1)	** < 0.0001**
3-mm choledochoscopy	0 (0)	98 (46.7)	** < 0.0001**
5-mm choledochoscopy	26 (100)	110 (52.4)	** < 0.0001**
Basket-in-catheter (BIC)	0 (0)	2 (1.0)	> 0.9999
*Biliary drainage (%)*			
T-tube	42 (17.7)	8 (3.3)	** < 0.0001**
Antegrade stent	140 (59.1)	12 (4.9)	** < 0.0001**
Transcystic drain	0 (0)	6 (2.5)	**0.0304**

Intra-operative data. *IQR* inter-quartile range; *LABEL* lithotripsy-assisted bile duct exploration by laparoendoscopy; *CBD* common bile duct

Bold text denotes *p* < 0.05

Post-operative outcome data for the two groups are displayed in Table 3. The overall stone clearance rate within the whole series was 96.3%. Successful stone clearance by LBDE was significantly higher within the *LATEST* group (98.8% vs 93.7%, *p* = 0.0034), and there was a trend towards increased post-operative ERCP use in the *pre-LATEST* group, although this did not reach significance (3.0% vs 0.8%, *p* = 0.1016). The reasons for failure of LBDE with their relative frequencies are summarised in Table 3. Although there was no difference in mortality (Clavien-Dindo V) between the two groups, there was significantly less minor (Clavien-Dindo I-II) and major (Clavien-Dindo III-IV) post-operative morbidity in the *LATEST* group. Specifically, there was less bile leak (5.5% vs 1.6%, *p* = 0.0262) and post-procedural pancreatitis (7.2% vs 0.8%, *p* = 0.0003) within the *LATEST* group. There were no CBD injuries secondary to laser lithotripsy in either group. As expected, median length of post-operative hospital stay was significantly reduced in the *LATEST* group (4 days vs 1 day, *p* < 0.0001).

**Table 3:** Pre-LATEST vs LATEST

	Pre-LATEST (*n* = 237)	LATEST (*n* = 244)	*p* value
Stone clearance by LBDE (%)	222 (93.7)	241 (98.8)	**0.0034**
Failure of LBDE (%)	15 (6.3)	3 (1.2)	
Conversion to open surgery	6 (2.5)	0 (0)	**0.0139**
Retained stones	9 (3.8)	3 (1.2)	0.0843
Post-operative ERCP (%)	7 (3.0)	2 (0.8)	0.1016
Median length of post-operative stay, days (IQR)	4 (2 – 9)	1 (1 – 3)	** < 0.0001**
*Complications (%)*			
Clavien-Dindo I-II	50 (21.1)	28 (11.5)	**0.0045**
Clavien-Dindo III-IV	13 (5.5)	4 (1.6)	**0.0262**
Clavien-Dindo V (30-day mortality)	3 (1.3)	0 (0)	0.1189
Bile leak	13 (5.5)	4 (1.6)	**0.0262**
Pancreatitis	17 (7.2)	2 (0.8)	**0.0003**
Bleeding	1 (0.4)	1 (0.4)	> 0.9999

Outcome data. *LBDE* laparoscopic bile duct exploration; *IQR *inter-quartile range; *ERCP* endoscopic retrograde cholangiopancreatography

Bold text denotes *p* < 0.05

## Discussion

At our institution, the adoption of LATEST in LBDE was associated with an increased stone clearance rate and a higher proportion of patients who received transcystic exploration, resulting in significantly improved post-operative outcomes. Choledocholithiasis is a common condition and this paradigm shift in practice has led to reduced post-operative morbidity (including bile leak and post-procedural pancreatitis) and shorter post-operative hospital stay.

During the evolution of LBDE at our institution over that last 23 years, we have changed practice and adapted the technique in order to increase the transcystic exploration rate. We recently proposed the concepts of *Biliary Surgery 2.0* and Leveraging Access to Technology and Enhanced Surgical Technique (LATEST) in LBDE [16, 17, 28]. *Leveraging access to technology* includes using thinner and more flexible choledochoscopes combined with fragmentation techniques such as laser or electrohydraulic lithotripsy. Prior to lithotripsy techniques, transcystic LBDE was limited to smaller CBD stones that were amenable to flushing into the duodenum or extraction via the cystic duct. In 2017, we published our initial results using holmium laser lithotripsy (HLL) during what we coined as the Lithotripsy-Assisted Bile duct Exploration by Laparoendoscopy (LABEL) technique [25]. Since then, we have demonstrated that using lithotripsy alone increased the transcystic exploration rate from 67 to 83% [26]. Without lithotripsy, we estimate that the ceiling of transcystic LBDE is somewhere around 60–70% [29–31]. More recently, we published a scoring tool (the ABCdE score) for PREdicting Lithotripsy Assistance during transcystic Bile duct Exploration by Laparoendoscopy (PRE-LABEL) [21, 32]. Over the last seven years, we have been routinely using single-use ultra-thin (~ 3 mm) choledochoscopes at our institution to facilitate transcystic LBDE [12]. Similarly, other authors have reported the use of ultra-thin choledochoscopes (with laser lithotripsy) to enable higher rates of transcystic exploration [33, 34]. *Enhanced surgical technique* refers to full mobilisation of the gallbladder followed by complete dissection of the cystic duct to the cysticocholedochal junction. The cystic duct is then retracted through the abdominal wall to create an optimal 90° cystic duct-common bile duct angle prior to attempting cannulation [12]. Enhanced surgical technique also refers to the trans-infundibular approach, which we have previously described, and is indicated when Calot’s triangle cannot be safely dissected due to a ‘frozen’ hepatic hilum secondary to severe inflammation and/or fibrosis [18].

**Fig. 4:**
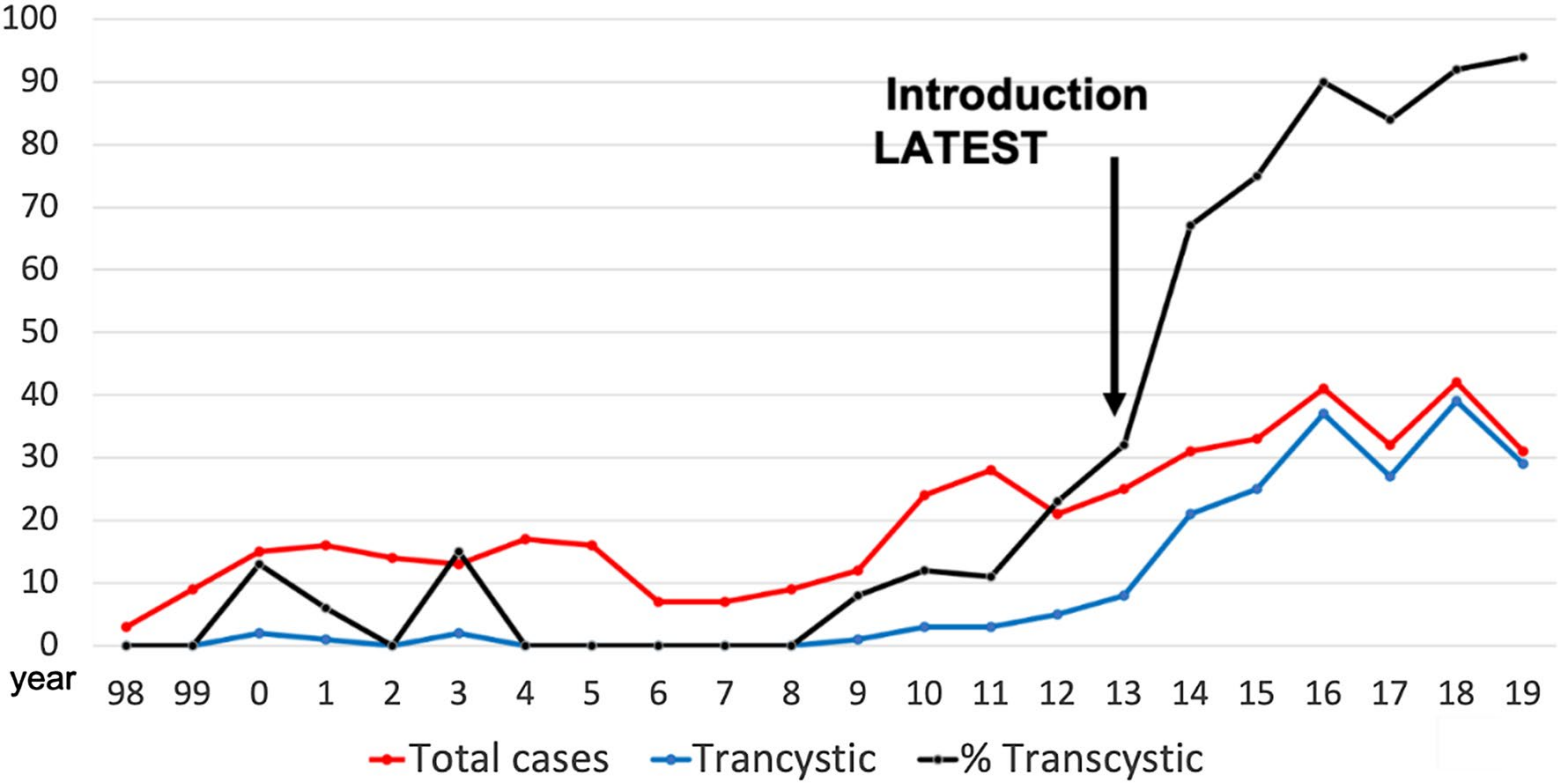
LBDE cases 1998 – 2019. Total number of cases (red), transcystic (blue) and % transcystic (black)

The benefits of transcystic, over transductal, LBDE have been well described at our institution and in the literature [12–15]. The post-operative morbidity associated with transcystic exploration is not too dissimilar to that of laparoscopic cholecystectomy. A recently published Society of American Gastrointestinal and Endoscopic Surgeons (SAGES) clinical spotlight review quoted a successful stone clearance rate via a transcystic approach of up to 71% [29, 30]. The findings from this study suggest that adhering to four principles, focused around technology and surgical technique, can increase the transcystic exploration rate to > 90% in an unselected group of patients. In the last 100 unselected patients, when all four LATEST principles were fully observed, the transcystic LBDE rate was 94%. The trend of transcystic LBDE at our institution over 20 years since starting the technique is represented in Fig. 4. A steep increase in transcystic exploration rates is evident after 2014, when the LATEST principles were implemented. Of the preceding 50 patients who underwent LBDE prior to LATEST, 28% were by the transcystic route, which is similar to the pooled data from randomised trials comparing LBDE to two-staged laparoendoscopic management of CBD stones (~ 32% transcystic) [5, 11]. Exploration of the bile duct transcystically not only leads to improved outcomes, but also lowers the threshold for choledochoscopy. An equivocal intra-operative cholangiogram often poses a difficult question to the surgeon: end the procedure accepting a small risk of a retained stone or explore the bile duct which more than likely will be normal. The transcystic approach swings the balance towards exploration and should be considered as a viable option if there is any diagnostic doubt. In the *LATEST* group, the negative exploration (negative choledochoscopy) rate was almost double compared to the *pre-LATEST* era, with these patients having a very low incidence of post-operative morbidity. The lower threshold to perform transcystic choledochoscopy in such equivocal cases should be weighed up against the extra cost of the disposable ultra-thin choledochoscope (range: £800-£1,800).

Our technique to correct the cysticocholedochal angle, as described, involves the following sequence: (1) dissection of Calot’s triangle to achieve the critical view of safety followed by ligation of the cystic artery (2) mobilisation of the gallbladder from the liver (3) complete skeletonization of the cystic duct to the cysticocholedochal junction (4) retraction of the infundibular-cystic duct junction through the abdominal wall (Fig. 3A-C). To our knowledge, this technique has not previously been described by other authors. However, in 1994, DePaula et al., described their technique of transcystic stone extraction (mainly using baskets under fluoroscopic control) with dissection of the cystic duct to the junction of the common bile duct without using cautery [35]. The authors specified that management through the cystic duct was considered the first option and choledochotomy was used for patients in which the cystic approach was not possible or unsuccessful, and achieved a transcystic stone clearance rate of 84%. Full dissection of the cystic duct, including the junction between the cystic duct and CBD has also been described by other authors [34]. Complete dissection of the cystic duct is key to the success of transcystic LBDE, particularly when it is not dilated. Correction of the cysticocholedochal angle will also facilitate proximal (intra-hepatic) choledochoscopy using the wiper blade manoeuvre.

Since the first choledochoscopy in the UK by Longland in 1975, the quality and availability of devices have substantially improved [36]. The inability to pass a choledochoscope through the cystic duct has been a cause of failure for transcystic LBDE in previous studies, largely due to the non-availability of thinner endoscopes [37, 38]. The use of an ultra-thin (~ 3 mm) choledochoscope will allow access to the CBD through a non-dilated cystic duct with minimal requirement for dilatation (Fig. 2A). We routinely use a 9.5–12F ureteral access sheath (Flexor Ureteral Access Sheath, Cook Medical, Bloomington, IN, USA) to intubate and gently dilate the cystic duct over a PTFE guidewire (0.035-inch diameter, 145-cm length, 3-cm flexible tip, Cook Medical, Bloomington, IN, USA), which then affords access for a 3-mm choledochoscope (Fig. 2B). Ido et al., published an early report of transcystic choledochoscopy in 1996 [39]. The authors were able to gain access to the CBD via the cystic duct in 65 out of 70 (93%) patients, mainly due to gentle dilatation of the cystic duct lumen with Maryland forceps and the use of an ultra-thin (3.1 mm) choledochoscope. Furthermore, when performing LBDE in the 1990s, Thompson and Tranter discovered that the proportion of patients undergoing transcystic exploration rose from 21 to 37% after the acquisition of a 3-mm choledochoscope in addition to a 5-mm endoscope [40]. More recently, Xia and colleagues have successfully demonstrated that use of an ultra-thin (< 3 mm) choledochoscope was instrumental in achieving high rates of transcystic LBDE [34]. An ultra-thin choledochoscope was used in 63.5% of patients and contributed to an overall transcystic LBDE success rate of 93.7%. In their study, holmium laser lithotripsy was also used in 32.2% of patients, demonstrating the synergistic action of ultra-thin choledochoscopy and lithotripsy to increase transcystic stone clearance, a finding confirmed by the results of the current study.

In 2019, we demonstrated that the use of lithotripsy techniques alone had increased our rate of successful transcystic LBDE to over 80% [26]. Fang et al., successfully performed 205 transcystic explorations using a 5-mm choledochoscope [41]. Their operative technique to gain access via the cystic duct was different to that described here. When the cystic duct was non-dilated (< 5 mm), a T-shaped incision of the cystic duct was made, with the stem of the ‘T’ extending towards the cysticocholedochal junction. Furthermore, the duct was routinely dilated using a balloon catheter. The reported bile leak rate within this group was 3.3%, which is higher than expected following transcystic LBDE (~ 1%). We would not advocate this manoeuvre as the T-shaped incision can easily extend to the bile duct (effectively performing a choledochotomy) either during the incision itself or when performing balloon dilatation. It is possible that this modified cystic duct incision could be avoided altogether if an ultra-thin choledochoscope was used. Nevertheless, FREDDY (frequency-doubled double-pulse neodymium/YAG) laser lithotripsy was required in 36.1% of patients, which otherwise would have necessitated choledochotomy and transductal extraction, resulting in failure of transcystic LBDE. The increased financial burden associated with laser lithotripsy is related to the one-off cost of the device (approximately £50,000) and the single-use of a laser fibre (£300-£500) [22].

The retrospective, non-random nature of this study is the primary limitation to generalising the results. In particular, the four principles of LATEST were not all implemented at exactly the same time. The earliest use of our preferred method to correct the cysticocholedochal angle was in May 2012, but was routinely used after 2014. The inaugural use of an ultra-thin choledochoscope at our institution was in October 2014. At that time, a reusable fibre-optic choledochoscope was used. The first attempted laser lithotripsy (LABEL procedure) was performed using the trans-infundibular approach (TIA) to the bile duct in February 2014. This date was chosen to define the LATEST era. Another major limitation of a ‘before and after’ study of a single series is that any learning curve associated with the procedure is likely to have an effect on outcomes. Even without any significant change to practice, the second half of a series will likely yield better outcomes when compared to the first half, which may contain part or all of the learning curve. This sample selection could have introduced bias, however, the cohort of patients from this study represents an ‘all-comers’ series who unselectively underwent LBDE at our institution. Despite a learning curve in transductal and transcystic LBDE, the former requiring advanced skills in laparoscopic suturing and the latter advanced skills in laparoendoscopy, this study demonstrates that four principles have been largely responsible for increasing the rate of transcystic LBDE, which is independent to its learning curve. Further research is required to determine the optimal treatment strategy for managing choledocholithiasis with concomitant gallstones. Most of the available randomised trial data is dated, with the LBDE arms mainly performing transductal clearance (from pooled analysis, less than one-third of patients underwent transcystic LBDE). Prospective randomised studies comparing contemporary (mainly transcystic) LBDE to alternative laparoendoscopic strategies (namely pre-operative ERCP followed by laparoscopic cholecystectomy and laparoscopic cholecystectomy with intra-operative ERCP) should be the focus of future research.

## Conclusion

Leveraging Access to Technology and Enhanced Surgical Technique describe four key factors that can be used when performing LBDE. The adoption of LATEST in LBDE is associated with an increased stone clearance and a higher transcystic exploration rate. It is well known that patients undergoing transcystic, rather than transductal, LBDE for choledocholithiasis with concomitant gallstones have significantly improved outcomes. Transcystic clearance rates over 90% can be achieved in high volume centres and the primary goal of surgeons undertaking LBDE should be to clear the bile duct transcystically.

## Supplementary Information

Below is the link to the electronic supplementary material.

Supplementary file1 (MP4 34843 KB)

Supplementary file2 (MP4 70000 KB)

Supplementary file3 (MP4 65110 KB)

Supplementary file4 (MP4 62086 KB)
